# Self-swabbing versus assisted swabbing for viral detection by qRT-PCR: the experience from SARS-CoV-2 based on a meta-analysis of six prospectively designed evaluations conducted in a UK setting

**DOI:** 10.1007/s10096-024-04866-z

**Published:** 2024-06-10

**Authors:** Tom Fowler, David Chapman, Matthias E. Futschik, Sarah A. Tunkel, Edward Blandford, Elena Turek, Olumide Kolade, Sergio Souza da Cunha, Andrew Dodgson, Paul Klapper, Malur Sudhanva, Lindsey Davies, Sue Hill, Susan Hopkins, Tim Peto

**Affiliations:** 1 UK Health Security Agency, 10 South Colonnade, Canary Wharf, London, E14 4PU UK; 2 William Harvey Research Institute and the Barts Cancer Institute, Queen Mary University of London, London, UK; 3 Deloitte MCS Ltd, London, UK; 4 School of Biomedical Sciences, Faculty of Health, University of Plymouth, Plymouth, UK; 5 University of Manchester, Manchester, UK; 6 King’s College Hospital NHS Foundation Trust, London, UK; 7 Health Protection Research Unit in Healthcare Associate Infections and Antimicrobial Resistance, National Institute for Health Research, Oxford, UK; 8 University of Oxford, Oxford, UK; 9 NHS England, London, UK

**Keywords:** COVID-19, Self-testing, Self-swabbing, PCR, United Kingdom, Testing programme

## Abstract

**Purpose:**

In April 2020, the UK Government implemented NHS Test and Trace to provide SARS-CoV-2 quantitative reverse transcription polymerase chain reaction (qRT-PCR) testing for the public, with nose-and-throat swabbing for samples performed by trained staff. Self-swabbing (SS) would allow rapid scale-up of testing capacity and access. Six studies were undertaken to determine whether SS was as effective for detecting SARS-CoV-2 as swabbing performed by trained staff.

**Methods:**

Six prospective studies were conducted between April–October 2020, using six swab/media combinations. Differences between assisted swabbing (AS) and SS were evaluated for concordance, positivity, sensitivity, cycle threshold (Ct) values and void rates. Statistical analysis was performed using 95% confidence intervals (CIs), paired t-tests and model-based methods.

**Results:**

Overall, 3,253 individuals were recruited (median age 37 years, 49% female), with 2,933 having valid paired qRT-PCR results. Pooled concordance rate was 98% (95% CI: 96%, 99%). Positivity rate differences for SS (8.1%) and AS (8.4%) and differences in pooled sensitivities between SS (86%; 95% CI: 78%, 92%) and AS (91%; 95% CI: 78%, 96%) were nonsignificant. Both types of swabbing led to pooled void rates below 2% and strongly correlated Ct values. Age, sex and previous swabbing experience did not have a significant impact on concordance or sensitivity.

**Conclusion:**

The UK adopted a policy to promote self-testing for SARS-CoV-2 based on data demonstrating equivalence of SS versus AS. Positive outcomes with SS are likely generalisable to testing for other respiratory pathogens, and we consider self-sampling and self-testing essential for future pandemic preparedness.

**Supplementary Information:**

The online version contains supplementary material available at 10.1007/s10096-024-04866-z.

## Introduction

As part of the coronavirus disease 2019 (COVID-19) pandemic response, the UK Government established National Health Service (NHS) Test and Trace to deliver the national testing programme [1–4], where quantitative reverse transcription polymerase chain reaction (qRT-PCR, hereafter ‘PCR’) testing for severe acute respiratory syndrome coronavirus 2 (SARS-CoV-2) was provided free to members of the public with or without (self-declared) symptoms. Initially, testing was conducted on nasal (mid-turbinate level, one nostril) and throat (both peri-tonsillar areas) swab samples at in-person testing sites by trained staff [5].

To control SARS-CoV-2 transmission, the testing service needed to scale-up to be available to anyone who self-declared symptoms. A key constraint was availability of trained staff to perform swabbing and, hence, number of appointment slots. A further access limitation was the need to attend testing sites in-person, which was impractical for many (due to shielding, immobility or transport issues). This health inequity was an unintended artefact of the testing service, which required mitigation.

One approach to addressing these challenges was to investigate whether individuals could collect samples themselves [6]. At the time, there was little evidence showing individuals could swab themselves to achieve accurate testing for SARS-CoV-2. Furthermore, in the early pandemic, global supply chain shortages of swabs and collection vials limited the capacity of the COVID-19 testing service.

As no single swab–vial combination was available at volumes to deliver the public health response, six prospective studies—with identical inclusion criteria, but different swab–vial combinations—were undertaken to assess the suitability of use as a self-sampling collection device across the range of devices available. In each study, individuals collected samples by themselves (hereafter, self-swabbing [SS]) and were then swabbed by a trained staff member (hereafter, assisted swabbing [AS]), allowing direct comparison between SS and AS. Here, we report results from a meta-analysis of the six service evaluation studies, to determine whether a difference in performance (measured by ability to detect SARS-CoV-2) between SS and AS could be observed.

## Materials and methods

### Data collection

Six service evaluation studies were conducted between April and October 2020 at three COVID-19 testing sites in the UK (Chessington, Manchester and Leicester) to compare the performance of SS versus AS for PCR testing. In this context, as was standard practice for routine PCR testing at the time.

Participants aged ≥ 18 years, were required to have read and understood printed SS instructions (provided in English) with illustrations and agreed to participate. Study designs are summarised in Table 1. A minimum study size was determined, so that the 95% confidence intervals (Cis) for estimated concordance rates between AS and SS (as defined in Supplementary Table 1) will fall within a 10% margin. More specifically, CIs were calculated using the Clopper-Pearson method and simulated for a varying number of participants and concordance rates. This led to a minimum number of required participants of 78 per study.

**Table 1:** Overview of the six service evaluation studies, including the demographic characteristics of the study populations

	Study 1	Study 2	Study 3	Study 4	Study 5	Study 6	Total	
Product	MW951S Sigma Virocult Kit	Medline MD202003 dry swab with vial of 0.85% saline	PROVIR Viral Transport Kit (TS/5–34 A)	E&O BM1673- M043-3 Vial + Medium, Alphalab SW1040	Combination kit with ISS PBT093 tube filled with 3 ml TF saline and Citotest 2122-0008 swab	BD Improve Medical Instruments 8,110,111 vial, 550,040 A dry swab	.	
Inclusion criteria	≥ 18 years old; read and understood self-swabbing instructions	≥ 18 years old; read and understood self-swabbing instructions	≥ 18 years old; read and understood self-swabbing instructions	≥ 18 years old; read and understood self-swabbing instructions	≥ 18 years old; read and understood self-swabbing instructions	≥ 18 years old; read and understood self-swabbing instructions	.	
Location/site	Chessington Regional Test Centre and UK Biocentre	Manchester Airport	Leicester (Birstall Park & Ride)	Leicester (Birstall Park & Ride)	Leicester (Birstall Park & Ride)	Leicester (Birstall Park & Ride)	.	
N (recruited)	97	395	689	492	1,005	575	**3,253**	
N (with PCR outcome)	90	348	654	458	892	491	**2,933**	
Median age (years); (min–max)	43(19–67)	40(18–81)	37(18–84)	36(18–81)	38(18–81)	35(18–84)	**37(18–84)**	
Sex: male (n,%)	4 (4)	113 (33)	286 (44)	212 (47)	447 (50)	239 (49)	**1,301 (44)**	
Sex: female (n,%)	9 (10)	117 (34)	362 (55)	243 (54)	445 (50)	252 (51)	**1,428 (49)**	
Sex: missing (n,%)	77 (86)	118 (34)	6 (1)	3 (1)	0	0	**204** **(7)**	
Previous swabbing experience (%)	39	20	23	20	56	n/a	**29**	

The original study names were: Study 1: SE-SWTC1/SSES; Study 2: SE-SWTC3/COMBI006; Study 3: TS5-34 A; Study 4: COMBI021; Study 5: COMBI031; and Study 6: COMBI045. PCR, polymerase chain reaction

A throat sample (both peri-tonsillar areas) and then a nasal sample (single nostril to the mid-turbinate level) were collected by participants using a single swab, without trained staff involvement [7]. This swab was placed by the participant into a fresh tube filled with viral transport medium and sealed/packaged and placed in collection boxes. Immediately afterwards, a further throat and nasal sample was collected using a fresh single swab, by a trained staff member. This was placed into a fresh tube filled with viral transport medium as for the SS sample. Although samples were collected by different people, care was taken to ensure handling was as similar as possible. Within studies, paired samples were collected using swabs and viral transport medium from the same manufacturer. Data on selfreported age, sex and previous (professional and self-) swabbing experience were collected on-site at time of attendance.

After sample collection, sealed tubes containing swabs and viral transport medium in collection boxes were batched and transported within 4 h to UK Biocentre, Milton Keynes, where all PCR testing was conducted [8]. For PCR testing, Thermofisher TaqPath COVID-19 CE-IVD PCR assays were used. These assays amplify specific regions (*ORF1ab*, *N* and *S* genes) of the SARS-CoV-2 genome and use a bacteriophage MS2 as internal control for PCR and extraction. In line with manufacturer’s instructions for use, a PCR test was deemed positive if the cycle threshold (Ct) value was < 40 for one or more target genes. The Ct value [8] was also reported and converted to viral concentrations (VCs) based on previous calibration [9]. Samples or PCR traces not meeting specified validity criteria (e.g., due to low sample volume, incorrect amplification curves or control probe not amplifying) were declared void. Processing technicians were not aware whether samples were collected by SS or AS.

### Statistical analysis

To compare outcomes between SS and AS, contingency tables were constructed for each study and the pooled dataset. Subsequently, concordance and Cohen’s kappa coefficients were calculated, alongside positive and negative percentage agreement (PPV/NPV). Definitions of statistical measures are provided in Supplementary Table 1. Contingency tables were further assessed using McNemar’s tests. Additionally, void and positivity rates for SS and AS were calculated. For sensitivity derivation, participants were regarded as COVID-19 positive if either swab or both swabs showed a positive outcome. 95% CIs for proportions were derived using the Clopper–Pearson (exact) method and proportions were compared using two-sided chi-squared tests with Yates’ continuity correction. Where p-values were calculated for all studies simultaneously, adjustment for multiple testing (*N* = 6) using the Holm method was performed. Ct values for the three target genes were averaged and compared between SS and AS using paired t-tests and Spearman correlation. The probability of a concordant positive result of SS was modelled by logistic regression with SS Ct value, age group (18–40, 41–60 and ≥ 61 years), sex and previous swabbing experience as independent variables. Finally, a random-effects meta-analysis of the six studies was performed applying the R package meta [10]. Pooled estimates of rate or proportions (concordance, sensitivity, void rates) and rate differences were obtained using the inverse variance method and the DerSimonian–Laird estimator for between-study variance [11]. This approach gives weights to each study, which are the inverse of the variance of the rate estimates. For calculation of the pooled sensitivity, for example, larger studies with respect to number of positive cases (such as study 6) were given more weight than smaller studies (such as study 1). 95% CI were derived using the Clopper–Pearson method and heterogeneity was reported based on the *I*^2^ statistics [12]. Statistical analyses were conducted in R (version 4.2.1).

## Results

Between April and October 2020, 3,253 individuals were recruited, of whom 211 withdrew consent after sample collection (Table 1 and Supplementary Fig. 1). Void results were recorded for 109 participants. Pooled void rates of SS and AS were 1.8% (95% CI: 0.9%, 3.7%) and 1.3% (95% CI: 0.6%, 3.0%) respectively (difference: 0.2%; 95% CI: −0.6%, 1.1%; Supplementary Fig. 2).

Valid paired PCR samples were recorded for 2,933 individuals included in further analysis. Participant age ranged 18–84 years, with median age of 37 years; individuals ≥ 61 years of age constituted 8% of the total population with available age information (Table 1). In the population with evaluable swabs, 49% of participants were females and 44% were males; no information on sex was available for 7% (Table 1). Full age and sex distributions of study participants are given in Supplementary Tables 2 and 3, respectively. Of the total recruited population, 29% had previous swabbing experience.

**Fig. 1:**
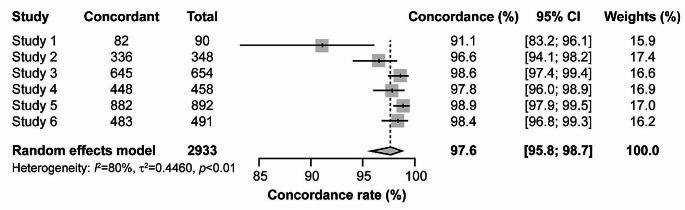
Forest plot of rate of concordance between SS and AS

Concordance between SS and AS was > 90% for all studies, and ≥ 95% in all except Study 1 (Fig. 1).

95% CI of single studies are shown as horizontal lines. Diamond indicates pooled estimate with left and right sides corresponding to lower and upper 95% CI.

AS, assisted swabbing; CI, confidence interval; SS, self-swabbing.

Pooled concordance across all studies was 98% (95% CI: 96%, 99%). Cohen’s kappa coefficients ranged 0.72–0.95, indicating substantial to almost perfect agreement (Supplementary Table 4). With respect to AS test results, SS showed a mean PPV of 87% and a mean NPV of 99%. Subgroup analysis indicated neither sex nor age had a statistically significant impact on concordance (Supplementary Tables 5 and 6).

**Fig. 2:**
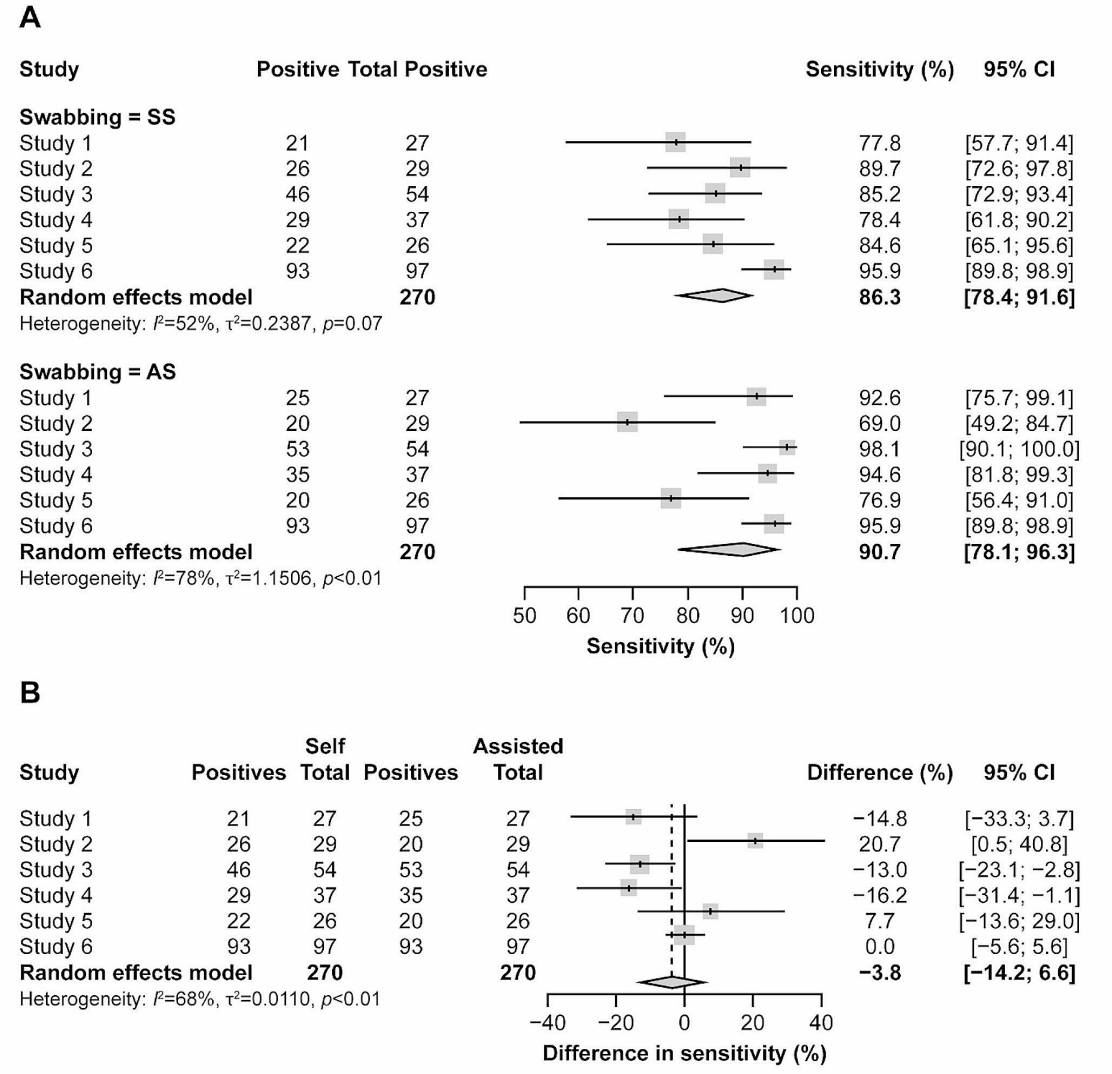
Forest plot comparing sensitivity in SARS-CoV-2 detection between SS and AS (**a**) Sensitivity of SS and AS across the six studies. (**b**) Differences in sensitivity between SS and AS. 95% CI of single studies are shown as horizontal lines. Diamond indicates pooled estimate with left and right sides corresponding to lower and upper 95% CI. Dashed line indicates pooled difference in sensitivity. AS, assisted swabbing; CI, confidence interval; SARS-CoV-2, severe acute respiratory syndrome coronavirus 2; SS, self-swabbing

Positivity rates for SS (8.1%) and AS (8.4%) were not statistically significantly different (*p* = 0.70) (Supplementary Table 7); however, 10% and 13% of positive tests for SS and AS were negative for the other approach. Assuming any positive outcome by SS and AS is a true positive result, we derived a pooled sensitivity of 86% (95% CI: 78%, 92%) for SS and 91% (95% CI: 78%, 96%) for AS (Fig. 2A).

Heterogeneity was observed between studies, with studies 2 and 5 indicating higher sensitivity of SS and studies 1, 3 and 4 indicating higher sensitivity of AS. However, the meta-analysis showed pooled difference in sensitivity was not statistically significant (− 3.8%; 95% CI: −14.2%, 6.6%; Fig. 2B).

**Fig. 3:**
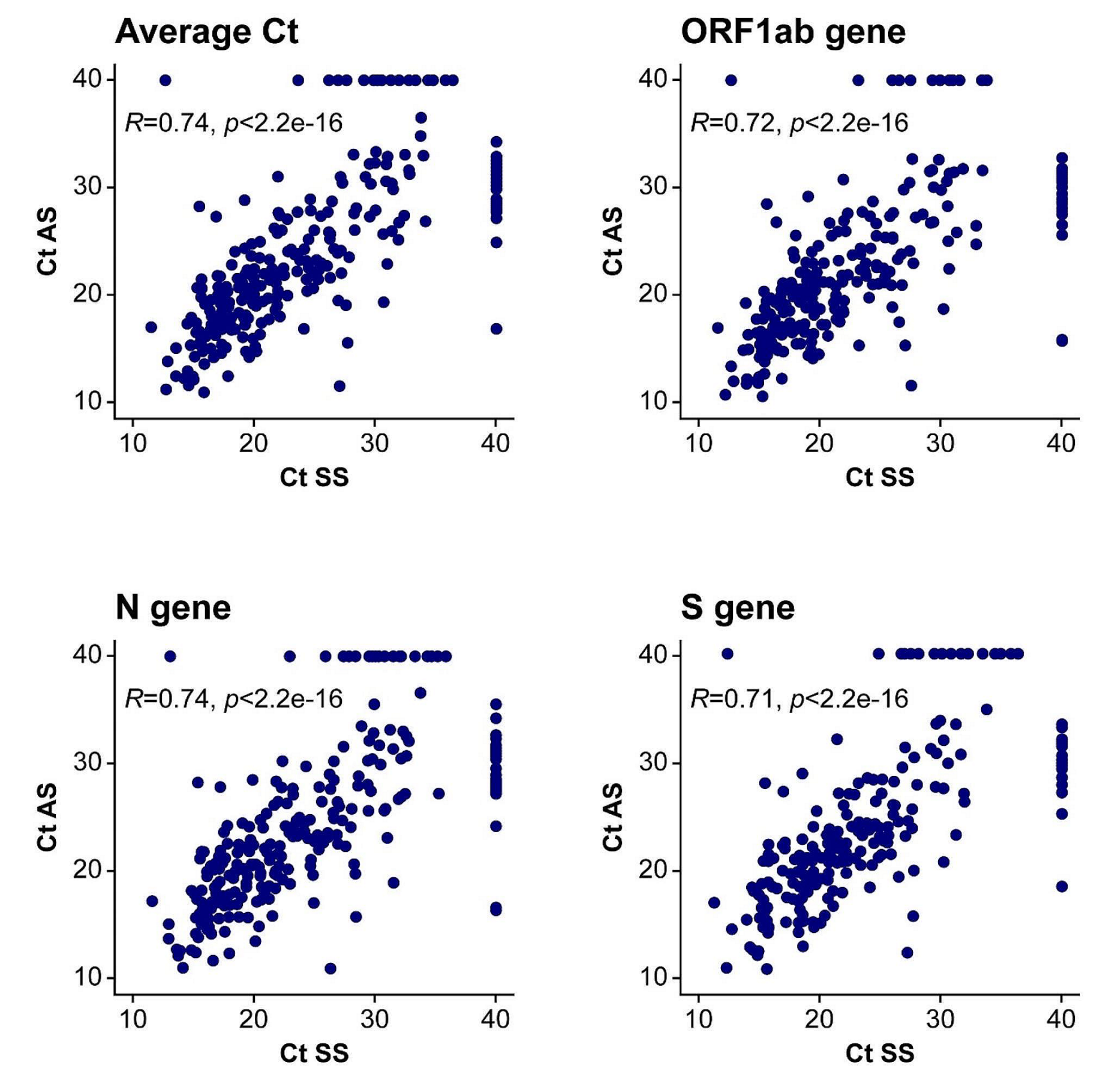
Scatterplots of Ct values measured in paired AS and SS samples For visualisation purposes, Ct values were set to 40 when SARS-CoV-2 was undetectable by qPCR in one of the paired samples. Thus, dots on the Ct = 40 horizontal or vertical lines represent samples which were positive either for the AS (vertical line) or SS (horizontal line) only. These values were not included for calculation of the Spearman correlation coefficient *R* and its significance. AS, assisted swabbing; Ct, cycle threshold; qPCR, quantitative polymerase chain reaction; SARS-CoV-2, severe acute respiratory syndrome coronavirus 2; SS, self-swabbing

A Spearman coefficient > 0.7 between AS and SS was observed for average Ct values and those for individual PCR target genes (Fig. 3).

Overall mean Ct values were 21.5 and 21.4 for SS and AS respectively (Supplementary Table 7) and paired Student’s *t*-test did not indicate statistically significant differences for the average Ct values or those of individual genes (Supplementary Fig. 3). Visualisation of Ct values of paired samples with only one positive test suggested the majority of discordant samples had high Ct values (Fig. 3). Of 33 positive AS samples with a paired negative SS sample, only two (6%) had a Ct value < 25; of 24 positive SS samples with a paired AS negative, only two (8%) had a Ct value < 25.

**Fig. 4:**
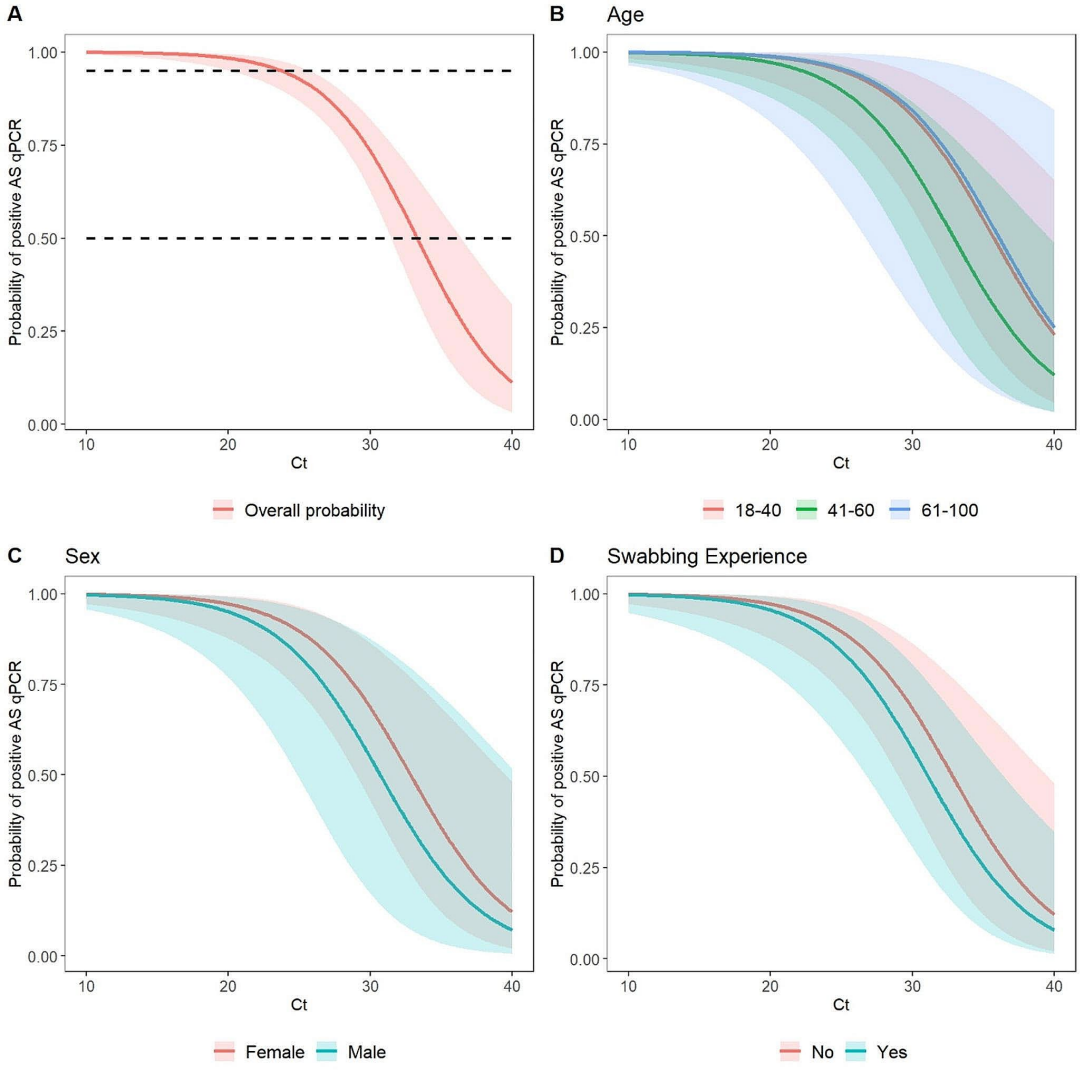
Predicted probability of a concordant positive AS-based SARS-CoV-2 test for a positive SS-based test, based on Ct value and stratified by age brackets (**B**), sex (**C**) and previous swabbing experience (**D**). Probabilities displayed were predicted based on the full cohort. Dashed lines indicate 50% and 95% probabilities (**A**). Probabilities were predicted for female participants with no previous swabbing experience (**B**), for participants 41–60 years of age and no previous swabbing experience (**C**), and for female participants 41–60 years of age (**D**). Shaded bands indicate the 95% CI. AS, assisted swabbing; CI, confidence interval; Ct, cycle threshold; qPCR, quantitative polymerase chain reaction; SARS-CoV-2, severe acute respiratory syndrome coronavirus 2; SS, self-swabbing

There was strong statistical evidence for the association between SS-based Ct value and positive AS outcome (*p* < 0.001), while age, sex and previous swabbing experience were not significant predictors of a positive AS-based outcome (Supplementary Table 8). The predicted probability of a positive AS outcome showed probability remained higher than 95% up to a Ct value of 20 and dropped below 50% only for Ct values > 30 (Fig. 4).

## Discussion

Data collected from six studies during the early COVID-19 pandemic in the UK were used to assess suitability of swab-vial combinations as SS devices within the COVID-19 testing programme. The high concordance of SS and AS regarding test results, positivity rate or Ct values based on over 2,900 participants suggested SS did not disproportionately miss individuals with lower viral concentration which, hypothetically, may have been characteristic of poorer first-time user technique. These findings supported decision-making regarding the use of SS during the COVID-19 pandemic.

Study populations were well-balanced, with similar proportions of males and females recruited overall. There was no statistically significant difference in concordance rates when stratified by sex. When our studies were conducted, emphasis was on testing people from the UK National Health Service, other key workers and their household contacts, most of whom were working age. Most participants were 18–40 years of age, with ~ 1/3 being 41–60 years of age, and < 10% were ≥ 61 years of age. These demographics align with participant populations in similar studies [13–15]. There was no statistically significant difference in concordance between age groups, which aligns with a previous study of South Korean patients that reported older age did not affect concordance between SS and AS results [16].

Sensitivity results reported here align with those published previously. A 2020 analysis among participants in Washington, USA, found sensitivities of 94.0% (97.5% CI: 83.8%, 100%) and 96.2% (97.5% CI: 87.0%, 100%) for participant-collected nasal and mid-turbinate samples, respectively [17], overlapping with the pooled sensitivity rates for SS (86%) and AS (91%) we observed. Other studies report sensitivities for participant-collected nasal and oropharyngeal samples of 80–99%, respectively [13, 14, 18–20].

We observed correlation of Ct value for SS vs. AS (*r* = 0.74) within the range reported previously. Two studies conducted in the USA in 2020 reported Pearson correlations from 0.78 to 0.86 between AS nasopharyngeal and SS nasal and mid-turbinate swabs [17, 18]. An analysis conducted in Bangladesh in 2021 found correlations of 0.82 and 0.81 for the COVID-19 *N* and *ORF1ab* genes, respectively, between AS nasopharyngeal samples and SS nasal samples [13]. Other studies conducted in India and Denmark have reported weaker correlations (0.356 and 0.4534, respectively) [15, 19]. We also observed most discordant samples had high Ct values for either the AS or SS sample. Similar associations between Ct values/viral concentration and SS sensitivity have been reported in other studies [13, 15–18].

While efficacy and accessibility of SS had been demonstrated for other respiratory illnesses before the pandemic [21], there was uncertainty this would translate to SARS-CoV-2. It is notable that at the start of the pandemic, there were no PCR respiratory sample collection kits approved for SS identified by the UK national testing programme. Our results demonstrated it is possible for individuals to reliably self-swab to detect SARS-CoV-2. This directly led to policy changes regarding SARS-CoV-2 testing, resulting in larger in-person testing centre throughput, opening of a home testing channel and a mass asymptomatic self-testing programme. However, results from these studies were not used to select swab/vial combinations for widespread use; in these six studies, assessed swab/vial combinations were designed for professional use and repurposed for self-use without undergoing extensive usability studies. Other swab/vial combinations had to pass additional laboratory, clinical and other compatibility tests to be included in the national testing programme. It should be noted that clear instructions must be provided in a format the person presenting for testing can understand, and this should be (and was) considered when planning wider implementation.

Technologies developed to help diagnose COVID-19 have been applied elsewhere, with growth in self-testing kit availability for various pathogens [22–25]. Our findings demonstrating comparability between SS and AS provide further support for the perspective that self-sampling and self-testing are likely to become an increasingly important healthcare component, outside of pandemic responses. Self-sampling has the potential to impact on clinical care, for example it could aid in managing conditions for vulnerable individuals by offering convenience, reducing exposure risks, and empowering individuals to take control of their health. Results supporting a paradigm shift in self-sampling have also been reported in other studies examining various swabbing approaches [13–19, 26]. Additionally, the global market for healthcare-associated self-testing is valued at ~ 20 billion US$ and expected to reach > 39 billion US$ by 2030 [27]. While our study showed SS can replace AS for a large part of the public in the case of COVID-19 testing, it remains essential from a regulatory perspective to validate reliability and performance of SS for future applications through comprehensive studies like ours. Taken alongside other changes in healthcare provision observed during the pandemic, including widespread telehealth uptake, this suggests an increasing shift to home-based paradigms of healthcare [28].

This meta-analysis has several strengths. To our knowledge, this is the largest analysis to date comparing performance of SS versus AS in a real-world setting, with > 2,900 participants. This participant volume enabled development of detailed sub-group analyses and model-based approaches to detect any factor which influenced the outcome measures. Additionally, we assessed for potential effects of previous swabbing experience in participants, enabling statistical analysis of any association between first-time use and testing performance.

There were several limitations of this meta-analysis. Participation used a convenience sampling approach of only asking site attendees to be tested, leading to study populations that were not representative of the general population, but instead of those willing and able to attend COVID-19 testing sites. Individuals unable to consent were not included, including those who could not understand English. There was an ongoing programme of improvement incorporating user feedback throughout the testing programme to improve swabbing instructions and other aspects of user experience. While this was received positively, although this may have impacted on the comparability of studies, there was no evidence this affected performance over time. The presence of an observer may have impacted on generalisability of the self-swabbing performance; however, observers were instructed not to interfere with the self-swabbing process, minimising this risk. This study took place in the context of the early pandemic phase, utilising rapidly implemented testing infrastructure whose primary purpose was public health testing. No information was collected for people who declined to participate. The impact of the order of testing was not examined.

## Conclusion

The results reported here support the use of SARS-CoV-2 SS as a viable alternative to AS and endorse the benefits of a broader self-testing strategy. Demonstrating that SS was a viable approach triggered a step change in the UK’s COVID-19 pandemic response, confirming mass testing is possible. Looking ahead, the positive results in these studies support the suggestion that self-sampling and self-testing are essential for pandemic preparedness and will become a standard requirement for healthcare services in general.

### Electronic supplementary material

Below is the link to the electronic supplementary material.

Supplementary Material 1

## Data Availability

Data collected for the study, including de-identified individual participant data and a data dictionary defining each field in the set, will be available from NHS Digital’s Data Access Request Service with publication of this Article. Details of how to apply for access to the data via the Data Access Request Service are provided at https://digital.nhs.uk/services/data-access-request-service-dars.

## Abbreviations

AS: Assisted swabbing
CI: Confidence interval
COVID-19: Coronavirus disease 2019
Ct: Cycle threshold
NHS: National Health Service
NPV: Negative percentage agreement
qRT-PCR: quantitative reverse transcription polymerase chain reaction
PPV: Positive percentage agreement
SARS-CoV-2: Severe acute respiratory syndrome coronavirus 2
SS: Self-swabbing
UK: United Kingdom
VC: Viral concentration
