# Supplementary material for: Self-swabbing versus assisted swabbing for viral detection by qRT-PCR: the experience from SARS-CoV-2 based on a meta-analysis of six prospectively designed evaluations conducted in a UK setting
Source: Eur J Clin Microbiol Infect Dis. 2024 Jun 10;43(8):1621–30. doi: 10.1007/s10096-024-04866-z (PMC11271363; doi:10.1007/s10096-024-04866-z)

# Supplementary Materials

**Self-swabbing versus assisted swabbing for viral detection by qRT-PCR: the experience from SARS-CoV-2 based on a meta-analysis of six prospectively designed evaluations conducted in a UK setting**

European Journal of Clinical Microbiology and Infectious Diseases

## Tom Fowler^a,b*^, David Chapman^c^, Matthias E. Futschik^a,d^, Sarah A Tunkel^a^, Edward Blandford^a^, Elena Turek^c^, Olumide Kolade^a^, Sergio Souza da Cunha^a^, Andrew Dodgson^a^, Paul Klapper^e^, Malur Sudhanva^f^, Lindsey Davies^a^, Sue Hill^a^, Susan Hopkins^a,g^, and Tim Peto^h^

## ^a^UK Health Security Agency, London, United Kingdom;

## ^b^William Harvey Research Institute and the Barts Cancer Institute, Queen Mary University of London, London, United Kingdom;

## ^c^Deloitte MCS Ltd, London, United Kingdom;

## ^d^University of Plymouth, School of Biomedical Sciences, Faculty of Health, Plymouth, United Kingdom;

## ^e^University of Manchester, Manchester, United Kingdom;

## ^f^Kings College Hospital NHS Foundation Trust, London, United Kingdom;

## ^g^Health Protection Research Unit in Healthcare Associate Infections and Antimicrobial Resistance, National Institute for Health Research, Oxford, United Kingdom;

## ^h^University of Oxford, Oxford, United Kingdom

*Corresponding author:

Tom Fowler

Email: tom.fowler2@ukhsa.gov.uk
Phone number: 07866213347
Postal address: UK Health Security Agency, 10 South Colonnade, Canary Wharf, London, E14 4PU, UK

## Supplementary Tables

### Supplementary Table 1: Definition of statistics.

|  |  | **AS** | |  |
| --- | --- | --- | --- | --- |
|  |  | **PCR +ve** | **PCR -ve** | **Total** |
| **SS** | **PCR +ve** | a | b | a+b |
|  | **PCR -ve** | c | d | c+d |
|  | **Total** | a+c | b+d | N=a+b+c+d |

**Concordance rate = (a + d) / N**

**Sensitivity (AS) = (a + b) / (a + b + c)**

**Sensitivity (SS) = (a + c) / (a + b + c)**

**Positive percentage agreement (SS) = a / (a + c)**

**Negative percentage agreement (SS) = d / (b + d)**

**Kappa = 2 x (a x d − c x b) / ( (a + b) x (b + d) + (a + c) x (c + d)**

Supplementary Table 2: Distribution of participants within the age brackets of 18–40 years, 41–60 years and 61 and more years. Percentages were calculated based on the number of participants with age information available. Ratio of rates (*RR*) is defined by the ratio of the observed percentage within an age bracket in the study and the corresponding percentage for the population in England, based on the 2021 Census data (<https://www.ons.gov.uk/census>). A RR larger than 1 indicates overrepresentation of the age group among study participants compared with the general population, while an *RR* smaller than 1 indicates underrepresentation.

| **Study** | ***N***  **18–40 yrs** | ***N***  **41–60 yrs** | ***N***  **61+ yrs** | ***N* with age information** | ***N***  **missing** | **Percentage (18–40 yrs)** | **Percentage**  **(41–60 yrs)** | **Percentage**  **(61–100 yrs)** | ***RR***  **(18–40 yrs)** | ***RR***  **(41–60 yrs)** | ***RR***  **(61–100 yrs)** |
| --- | --- | --- | --- | --- | --- | --- | --- | --- | --- | --- | --- |
| **Study 1** | 6 | 5 | 2 | 13 | 77 | 46% | 38% | 15% | 1.21 | 1.17 | 0.53 |
| **Study 2** | 120 | 98 | 12 | 230 | 118 | 52% | 43% | 5% | 1.37 | 1.29 | 0.18 |
| **Study 3** | 371 | 220 | 55 | 646 | 8 | 57% | 34% | 9% | 1.51 | 1.03 | 0.29 |
| **Study 4** | 263 | 159 | 32 | 454 | 4 | 58% | 35% | 7% | 1.52 | 1.06 | 0.24 |
| **Study 5** | 487 | 330 | 75 | 892 | 0 | 55% | 37% | 8% | 1.44 | 1.12 | 0.29 |
| **Study 6** | 304 | 158 | 29 | 491 | 0 | 62% | 32% | 6% | 1.63 | 0.98 | 0.2 |
| **Total** | 1,551 | 970 | 205 | 2,726 | 207 | 57% | 36% | 8% | 1.5 | 1.08 | 0.26 |

**Supplementary Table 3: Sex distribution of participants**. Percentages were calculated based on the number of participants with sex information available. Ratio of rates (*RR*) is defined by the ratio of the observed percentage with sex information available in the study and the corresponding percentage for the population in England, based on the 2021 Census data (<https://www.ons.gov.uk/census>). A *RR* larger than 1 indicates overrepresentation of the sex among study participants compared with the general population, while an *RR* smaller than 1 indicates underrepresentation.

| **Study** | **Male** | **Female** | **N with sex information** | ***N* missing** | **Percentage Male** | **Percentage Female** | ***RR* Male** | ***RR* Female** |
| --- | --- | --- | --- | --- | --- | --- | --- | --- |
| **Study 1** | 4 | 9 | 13 | 77 | 31% | 69% | 0.62 | 1.37 |
| **Study 2** | 113 | 117 | 230 | 118 | 49% | 51% | 0.99 | 1.01 |
| **Study 3** | 286 | 362 | 648 | 6 | 44% | 56% | 0.89 | 1.11 |
| **Study 4** | 212 | 243 | 455 | 3 | 47% | 53% | 0.94 | 1.06 |
| **Study 5** | 447 | 445 | 892 | 0 | 50% | 50% | 1.01 | 0.99 |
| **Study 6** | 239 | 252 | 491 | 0 | 49% | 51% | 0.98 | 1.02 |
| **Total** | 1,301 | 1428 | 2,729 | 204 | 48% | 52% | 0.96 | 1.04 |

**Supplementary Table 4: Confusion matrices: concordance rate observed and expected by chance and other agreement statistics.** Positive and negative agreement was calculated for SS with respect to AS.

|  | **Results of the PCR according to mode of collection (swabbing)** | | | **Agreement statistics** | | | | |
| --- | --- | --- | --- | --- | --- | --- | --- | --- |
| **Study** | **SS** | **AS** | | **Proportion of agreement** | | | | **Kappa** |
|  |  | **+ve** | **-ve** | **Concordance by chance** | **Concordance observed** | **Positive**  **percentage**  **agreement** | **Negative**  **Percentage**  **agreement** |  |
| **Study 1** | **+ve** | 19 | 2 | 61.9% | 91.1% | 76.0% | 96.9% | 0.77 |
|  | **-ve** | 6 | 63 |  |  |  |  |  |
| **Study 2** | **+ve** | 17 | 9 | 87.6% | 96.6% | 85.0% | 97.3% | 0.72 |
|  | **-ve** | 3 | 319 |  |  |  |  |  |
| **Study 3** | **+ve** | 45 | 1 | 86.0% | 98.6% | 84.9% | 99.8% | 0.90 |
|  | **-ve** | 8 | 600 |  |  |  |  |  |
| **Study 4** | **+ve** | 27 | 2 | 87.0% | 97.8% | 77.1% | 99.5% | 0.83 |
|  | **-ve** | 8 | 421 |  |  |  |  |  |
| **Study 5** | **+ve** | 16 | 6 | 95.4% | 98.9% | 80.0% | 99.3% | 0.76 |
|  | **-ve** | 4 | 866 |  |  |  |  |  |
| **Study 6** | **+ve** | 89 | 4 | 69.3% | 98.4% | 95.7% | 99.0% | 0.95 |
|  | **-ve** | 4 | 394 |  |  |  |  |  |
| **Overall** | **+ve** | 213 | 24 | 84.9% | 98.1% | 86.6% | 99.1% | 0.87 |
|  | **-ve** | 34 | 2,663 |  |  |  |  |  |

**Supplementary Table 5:** **Concordance rates stratified by sex.** For study 1, CI for percentage point difference and statistical significance was not calculated due to low sample numbers.

| **Study** | **Male** | | **Female** | | **Difference** | | |
| --- | --- | --- | --- | --- | --- | --- | --- |
|  | **Concordance rate (%) [95% CI]** | ***N*** | **Concordance rate (%) [95% CI]** | ***N*** | **%** | ***p*** | **Adj. *p*** |
| Study 1 | 100.0 [39.8, 100.0] | 4 | 100.0 [66.4, 100.0] | 9 | 0 | - | - |
| Study 2 | 96.5 [91.2, 99.0] | 113 | 94.0 [88.1, 97.6] | 117 | 2.4 [−3.9, 8.8] | 0.58 | 1 |
| Study 3 | 99.0 [97.0, 99.8] | 286 | 98.3 [96.4, 99.4] | 362 | 0.6 [−1.5, 2.7] | 0.75 | 1 |
| Study 4 | 98.1 [95.2, 99.5] | 212 | 97.5 [94.7, 99.1] | 243 | 0.6 [−2.5, 3.7] | 0.92 | 1 |
| Study 5 | 98.4 [96.8, 99.4] | 447 | 99.3 [98.0, 99.9] | 445 | −0.9 [−2.5, 0.7] | 0.34 | 1 |
| Study 6 | 97.9 [95.2, 99.3] | 239 | 98.8 [96.6, 99.8] | 252 | −0.9 [−3.6,1.8] | 0.67 | 1 |
| **Total** | **98.2** [**97.4, 98.9]** | **1,301** | **98.2** [**97.4, 98.9]** | **1,428** | **0.0** [**−1.0, 1.0]** | **1.00** |  |

| Study | Age 61+ yrs | | | | | | Age 41–60 yrs | | | | | Age 18–40 yrs | | | | | |
| --- | --- | --- | --- | --- | --- | --- | --- | --- | --- | --- | --- | --- | --- | --- | --- | --- | --- |
|  | **Concordance rate (%)**  **[95% CI]** | ***N*** | **Diff. (%) 61+ yrs vs rest [95% CI]** | ***p*** | **Adj. *p*** | **Concordance rate (%)  [95% CI]** | | ***N*** | **Diff. (%) 41–60 yrs vs rest [95% CI]** | ***p*** | **Adj. *p*** | | **Concordance rate (%)  [95% CI]** | ***N*** | **Diff. (%) 18–40 yrs vs rest [95% CI]** | ***p*** | **Adj. *p*** |
| Study 1 | 100.0  [15.8, 100.0] | 2 | 0.0 | - | - | 100.0  [47.8, 100.0] | | 5 | 0.0 | - | - | | 100.0  [54.1, 100.0] | 6 | 0.0 | - | - |
| Study 2 | 83.3  [51.6, 97.9] | 12 | −12.5  [−38.2, 13.1] | 0.20 | 0.99 | 94.9  [88.5, 98.3] | | 98 | −0.6  [−6.7, 5.6] | 1.00 | 1.00 | | 96.7  [91.7, 99.1] | 120 | 3.0  [−3.4, 9.5] | 0.44 | 1.00 |
| Study 3 | 100.0  [93.5, 100.0] | 55 | 1.5  [−0.5, 3.5] | 0.75 | 1.00 | 98.6  [96.1, 99.7] | | 220 | 0.0  [−1.9, 2.0] | 1.00 | 1.00 | | 98.4  [96.5, 99.4] | 371 | −0.5  [−2.6, 1.6] | 0.82 | 1.00 |
| Study 4 | 96.9  [83.8, 99.9] | 32 | −1.0  [−8.2, 6.2] | 1.00 | 1.00 | 95.6  [91.1, 98.2] | | 159 | −3.4  [−7.3, 0.5] | 0.04 | 0.18 | | 99.2  [97.3, 99.9] | 263 | 3.4  [−0.1, 6.9] | 0.03 | 0.16 |
| Study 5 | 100.0  [95.2, 100.0] | 75 | 1.2  [−0.3, 2.7] | 0.70 | 1.00 | 98.8  [96.9, 99.7] | | 330 | −0.1  [−1.7, 1.5] | 1.00 | 1.00 | | 98.8  [97.3, 99.5] | 487 | −0.2  [−1.8, 1.4] | 0.98 | 1.00 |
| Study 6 | 100.0  [88.1, 100.0] | 29 | 1.7  [−1.2, 4.7] | 1.00 | 1.00 | 96.2  [91.9, 98.6] | | 158 | −3.2  [−6.8, 0.4] | 0.03 | 0.13 | | 99.3  [97.6, 99.9] | 304 | 2.6  [−0.6, 5.7] | 0.07 | 0.29 |
| TOTAL | 98.5  [95.8, 99.7] | 205 | 0.3  [−1.7, 2.3] | 0.95 |  | 97.4  [96.2, 98.3] | | 970 | −1.3  [−2.5, −0.1] | 0.02 |  | | 98.7  [98.0, 99.2] | 1,551 | 1.1  [0.0, 2.2] | 0.05 |  |

**Supplementary Table 6:** **Concordance rates stratified by age.** For study 1, CI for percentage point difference and statistical significance was not calculated due to low sample numbers.

| **Outcome** | **Study 1** | **Study 2** | **Study 3** | **Study 4** | **Study 5** | **Study 6** | **Overall** |
| --- | --- | --- | --- | --- | --- | --- | --- |
| **Positivity rate** (%) |  |  |  |  |  |  |  |
| SS | 23.3% | 7.5% | 7.0% | 6.3% | 2.5% | 18.9% | 8.1% |
| AS | 27.8% | 5.7% | 8.1% | 7.6% | 2.2% | 18.9% | 8.4% |
| Difference | −4.4% | 1.7% | −1.1% | −1.3% | 0.2% | 0.0% | −0.3% |
| *p* value | 0.61 | 0.45 | 0.53 | 0.52 | 0.88 | 1 | 0.70 |
| Adjusted *p* value | 1 | 1 | 1 | 1 | 1 | 1 |  |
| **Ct values** |  |  |  |  |  |  |  |
| SS, mean (SD) | 19.8 (4.3) | 21.8 (5.6) | 21.9 (5.9) | 21.2 (5.4) | 23.4 (6.6) | 21.4 (4.7) | 21.5 (5.3) |
| AS, mean (SD) | 23.1 (5.8) | 23.4 (5.1) | 20.6 (5.5) | 21.0 (5.7) | 23.6 (6.5) | 20.8 (4.7) | 21.4 (5.3) |
| Difference [95%] | −3.2 [−5.3, −1.2] | −1.6 [−2.9, −0.4] | 1.4 [0.2, 2.5] | 0.1 [−0.7, 0.9] | −0.3 [−2.2, 1.7] | 0.5 [−0.2, 1.3] | 0.1 [−0.4, 0.6] |
| *p* value | 0.004 | 0.022 | 0.021 | 0.761 | 0.785 | 0.154 | 0.71 |
| Adjusted *p* value | 0.024 | 0.105 | 0.105 | 1 | 1 | 0.462 |  |

**Supplementary Table 7: Overall agreement in the proportion of positive PCR results between SS and AS specimens.** Mean Ct values were calculated for participants with both AS and SS being positive.

| **Characteristic** | **Univariable regression** | | | | **Multivariable regression** | | |
| --- | --- | --- | --- | --- | --- | --- | --- |
|  | **N** | **OR** | **95% CI** | ***p* value** | **OR** | **95% CI** | ***p* value** |
| **Ct SS** | 237 | 0.74 | 0.66, 0.82 | <0.001 | 0.76 | 0.65, 0.86 | <0.001 |
| **Age bracket** | 215 |  |  |  |  |  |  |
| 18–40 yrs |  | — | — |  | — | — |  |
| 40–60 yrs |  | 0.62 | 0.25, 1.53 | 0.3 | 0.46 | 0.11, 1.78 | 0.3 |
| 60–100 yrs |  | 0.92 | 0.11, 7.92 | >0.9 | 1.11 | 0.11, 25.7 | >0.9 |
| **Sex** | 215 |  |  |  |  |  |  |
| Female |  | — | — |  | — | — |  |
| Male |  | 1.43 | 0.58, 3.50 | 0.4 | 0.55 | 0.12, 2.44 | 0.4 |
| **Previous swabbing**  **experience** | 138 |  |  |  |  |  |  |
| No |  | — | — |  | — | — |  |
| Yes |  | 0.56 | 0.21, 1.50 | 0.2 | 0.62 | 0.18, 2.20 | 0.5 |

**Supplementary Table 8: Univariable and multivariable logistic regression of AS test outcome.** N: Number of samples included in univariable logistic regression. OR: Odds ratio.

## Supplementary Figures

**Supplementary Figure 1: STARD flow chart.**


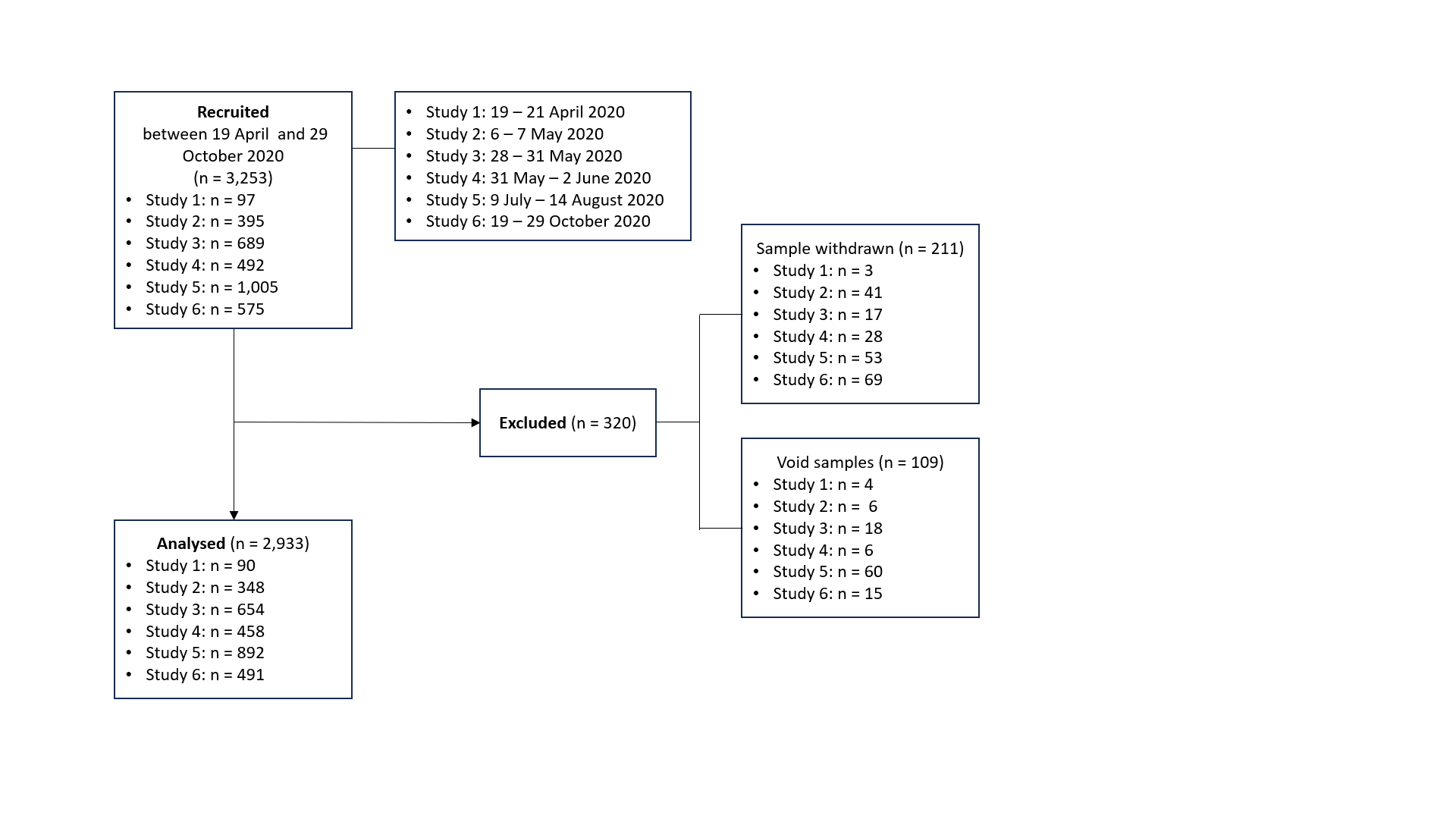


**Supplementary Figure 2: Forest plot of void rates.** (**A**) Void rates of SS and AS across the six studies. (**B**) Differences in void rates between SS and AS. 95% CI of single studies are shown as horizontal lines. Diamond indicates pooled estimate with left and right sides corresponding to lower and upper 95% CI. Dashed line indicates pooled difference in void rate.


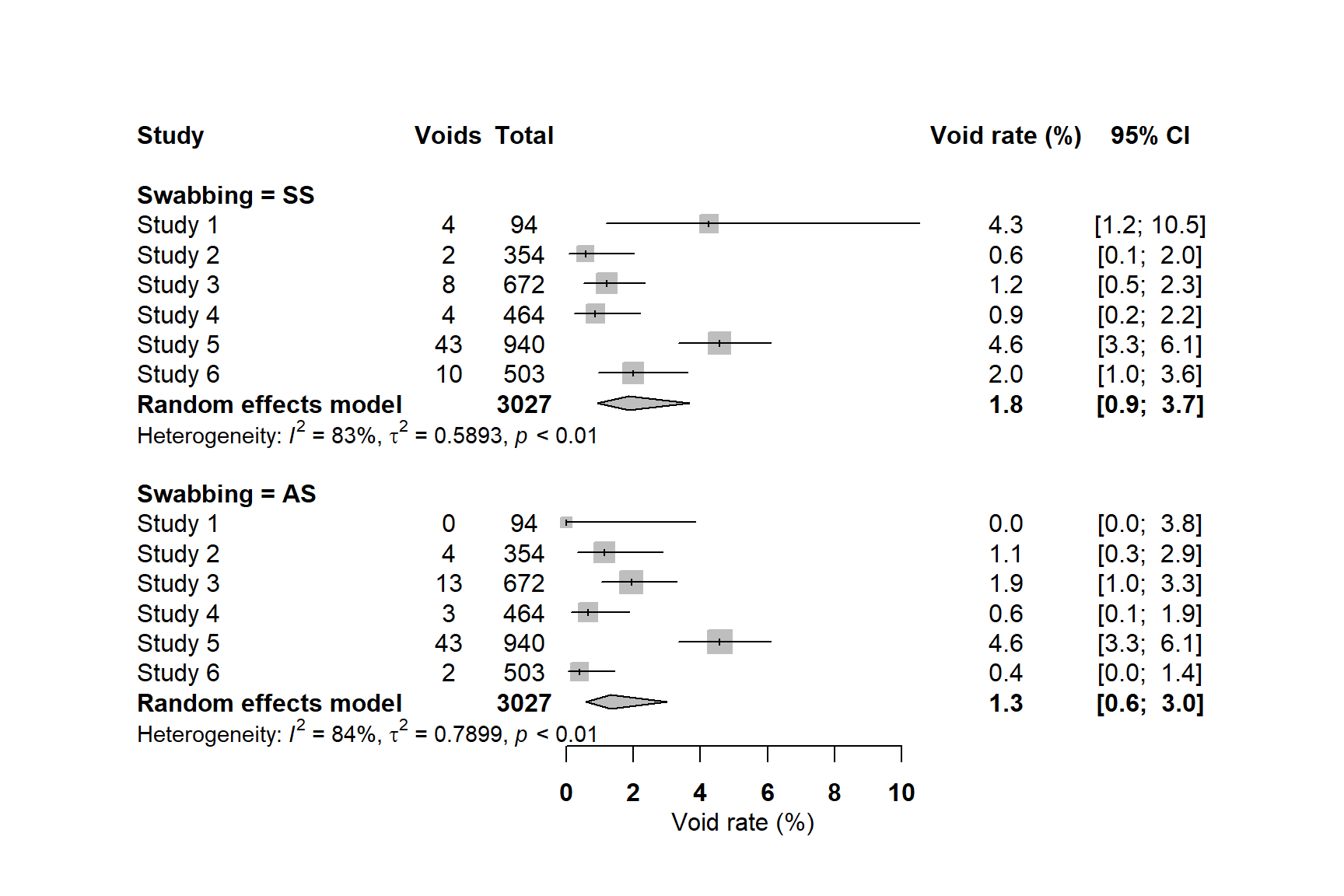

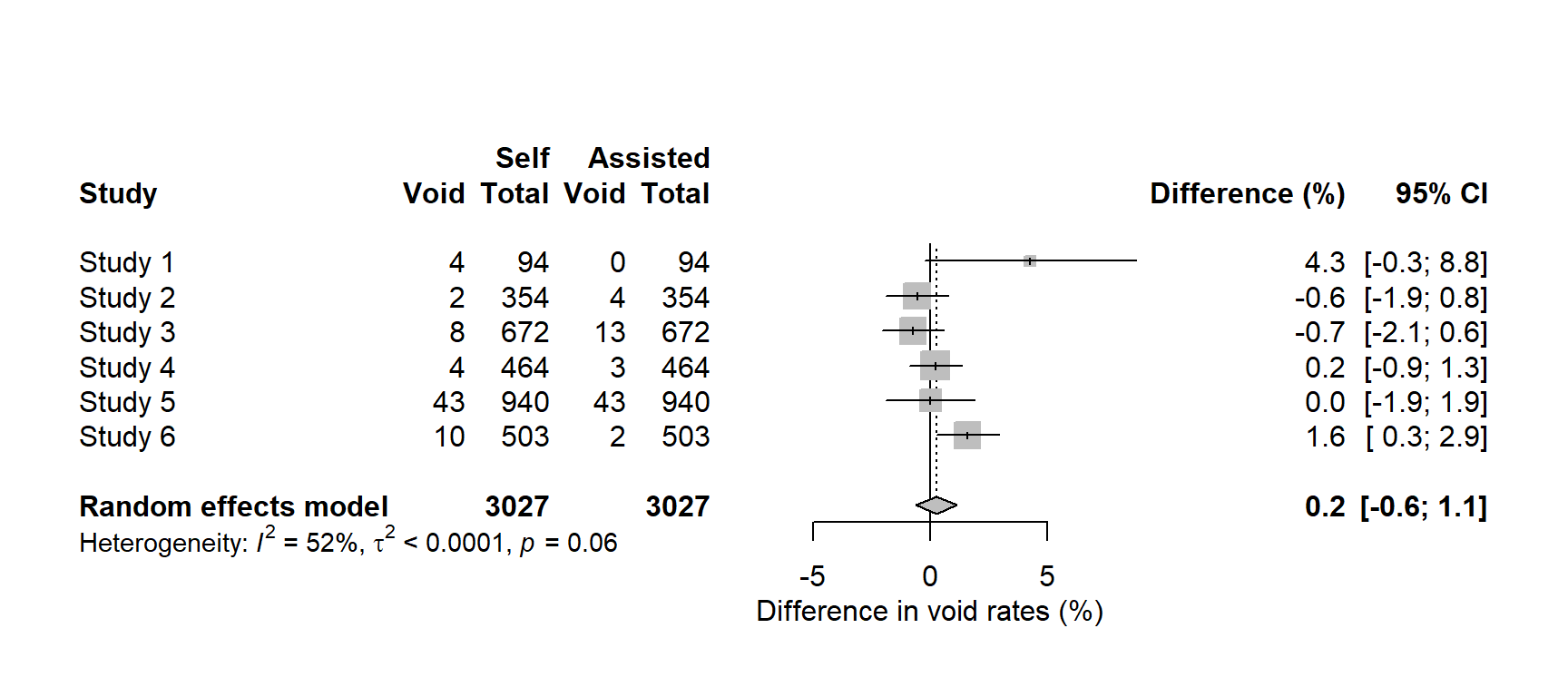


**B**

**A**

**Supplementary Figure 3: Distribution of Ct values of AS and SS samples.** Box and whiskers plots are displayed for **A**) averaged Ct values, and Ct values of **B**) Orf1ab gene, **C**) N gene and **D**) S gene for paired positive tests. The lower and upper hinges correspond to the first and third quartiles. Statistical significance (*p* values) shown for differences in Ct values between sampling types are based on paired Student’s t-test.


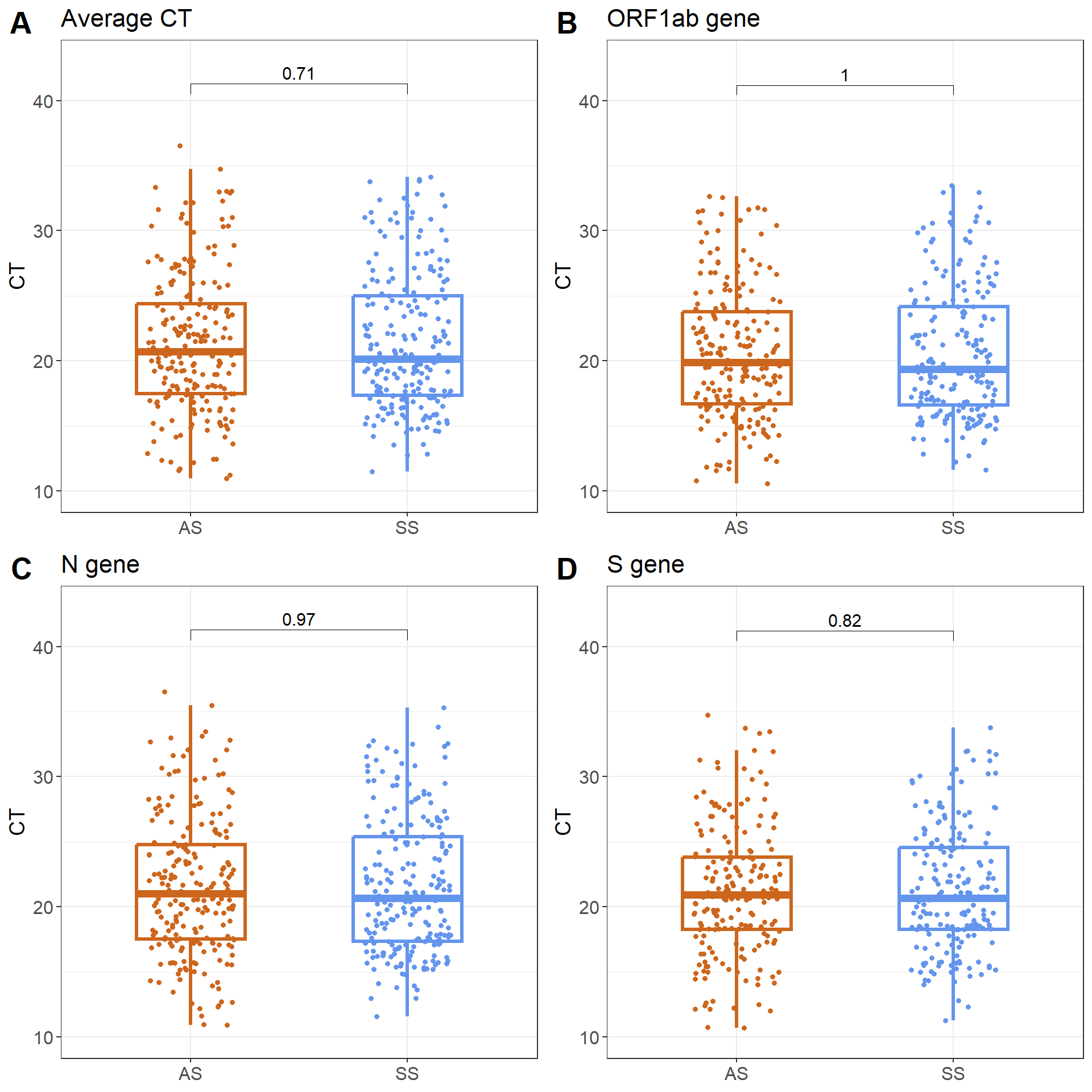

Supplement: Supplementary file 1 — Supplementary Material 1 [file 10096_2024_4866_MOESM1_ESM.docx]
